# Unlocking the Therapeutic Potential: Harnessing miR-125a-5p To Enhance Autophagy and Apoptosis in Pancreatic Cancer through Targeting STAT3

**DOI:** 10.7150/jca.97102

**Published:** 2024-07-16

**Authors:** Lujuan Pan, Zongshuai Qin, Qinghong Zhou, Pin Zheng, Hua Li, Xihan Zhou, Yueqiu Qin

**Affiliations:** 1 The First Affiliated Hospital of Jinan University, Jinan University, Guangzhou, Guangdong Province, China.; 2 Department of Gastroenterology, Affiliated Hospital of Youjiang Medical University for Nationalities, Baise, Guangxi Province, China.; 3 Key Laboratory of Tumor Molecular Pathology of Baise, Baise, Guangxi Province, China.; 4 Department of General Surgery, Affiliated Hospital of Youjiang Medical University for Nationalities, Baise, Guangxi Province, China.

**Keywords:** autophagy, apoptosis, miR-125a-5p, pancreatic cancer, STAT3

## Abstract

**Objectives:** miR-125a-5p's role in various cancers has been recognized, yet its specific function in pancreatic cancer (PCa) demands further exploration. This study aimed to reveal the potential function of miR-125a-5p in PCa.

**Methods:** With publicly available databases, we explored the expression pattern and prognostic relevance of miR-125a-5p and STAT3 in PCa. We measured miR-125a-5p levels in PCa tissues, plasma and cell lines using RT-qPCR. To assess functional effects, PANC-1 cells were transfected with miR-125a-5p mimics and inhibitors, as well as siRNA-STAT3 and STAT3 vectors. Cell proliferation was estimated using Cell Counting Kit-8, while autophagy and apoptosis were examined by transmission electron microscopy and TUNEL assay, respectively. Western blot analysis was also performed to detect proteins associated with autophagy and apoptosis. The regulatory relationship of miR-125a-5p on STAT3 was verified using a dual luciferase reporter assay. The influence of miR-125a-5p on tumor development was evaluated in xenograft models.

**Results:** Decreased expression of miR-125a-5p was found in PCa samples, and low expression of miR-125a-5p was associated with a poorer prognosis in PCa patients. Functional assays indicated miR-125a-5p suppressed cell growth while enhancing apoptosis and autophagy in PCa cells. STAT3 represents a specific target of miR-125a-5p, inhibiting STAT3 reversed the inhibitory effect of overexposed miR-125a-5p. Additionally, miR-125a-5p significantly restrained tumor development in mice.

**Conclusions:** miR-125a-5p functions as a tumor suppressor in PCa by targeting STAT3, thereby inducing autophagy and apoptosis. Its regulatory role underscores its potential as a valuable biomarker for PCa diagnosis and therapy, warranting further clinical investigation.

## Introduction

Pancreatic cancer (PCa) is recognized as one of the most fatal neoplasms worldwide^1^. Recent data indicate a progressive rise in both incidence and death rates associated with this malignancy annually^2^. Despite the advancements in detection and treatment strategies for PCa, the specific mechanistic underpinnings of oncogenesis and tumor growth of PCa are still unelucidated. Hence, it is imperative to fully delineate the complex molecular mechanistic underpinnings driving PCa for the identification of potential therapeutic targets against this malignancy.

MicroRNAs (miRNAs) represent a group of short, endogenously produced RNAs that do not code for proteins, typically comprising around 22 nucleotides^3^. Extensive evidence has highlighted the interaction between miRNAs and the 3′ zone in mRNAs, with miRNAs impeding the expression of downstream target genes through post-transcriptional mechanisms^4^, ^5^. As modulators of mRNA, miRNAs have been implicated in modulating a diverse array of biological functions and impart prominent effects on the initiation and progression of tumors^6^. A growing body of research has confirmed the diminishment of miR-125a-5p levels across a wide range of malignant tumors, such as gastric^7^, lung^8^, and cervical cancers^9^. The biological relevance of miR-125a-5p on restraining the proliferative, invasive, and migratory capacities suggests its potential as a tumor-suppressive target in PCa^10^. Nevertheless, the exact mechanistic underpinnings behind the functional relevance of miR-125a-5p in PCa remain incompletely understood.

Autophagy is a dynamic cellular function that degrades abnormal cellular components and maintains cellular homeostasis. Autophagy plays a dual role in tumors, either promoting or inhibiting tumorigenesis^11^. Accumulating studies have revealed that miR-143-5p and miR-24-3p drive the apoptotic and autophagic capacity in PCa cells, thereby contributing to a suppressive impact on the growth of tumor cells^12^, ^13^. Apoptosis is a regulated process through which the body eliminates damaged or redundant cells in an orderly manner, characterized by cellular changes such as condensation, nuclear fragmentation, and nucleolysis. A BCL-2 inhibitor has been approved for treating lymphoma by targeting and modulating this apoptotic pathway^14^.

It is intriguing to note the potential involvement of signal transducer and activator of transcription (STAT) proteins, a common family of transcription factors, in a diverse array of biological processes^15^. This family consists of STAT1-4, STAT5A/B, and STAT6 in the human genome. Notably, elevated levels of STAT3 in a range of cancers have been linked to dismal prognostic outcomes^15^. Shukla SK *et al.* have pinpointed that STAT3 contributes to the acceleration of malignancy and progression of PCa; thus, the targeted inhibition of STAT3 presents a viable therapeutic approach for managing PCa^16^. Prior research has also uncovered that miR-125a-5p curtails the proliferative and migratory potential by diminishing STAT3 activation in esophageal squamous cell, breast, and lung carcinomas^8^, ^17^, ^18^. Nevertheless, the capacity of miR-125a-5p to specifically target STAT3, then arrest PCa growth has not been determined.

Our investigation revealed a notable decline in the miR-125a-5p levels in PCa tissues, plasma, and cell lines, and found a positive correlation between its high expression and improved prognosis. Functionally, miR-125a-5p demonstrated antioncogenic effects by impeding the proliferative potential and fostering autophagy and apoptosis through direct interaction with STAT3. These findings emphasize the significance of miR-125a-5p as a promising target for the detection and therapeutic intervention of PCa.

## Materials and methods

### Bioinformatics analysis

The GSE24279, GSE41369, and GSE114778 datasets were accessed from the Gene Expression Omnibus (GEO) platform. Data on bulk RNA sequencing alongside clinical profiles of individuals with PCa were sourced from The Cancer Genome Atlas (TCGA) website. The expression and prognostic efficiency of miR-125a-5p and STAT3 on PCa were examined and graphically presented via the Xiantao platform (https://www.xiantao.love/).

### Clinical specimen collection

PCa tissues and matched adjacent non-neoplastic tissue specimens were obtained from eight individuals diagnosed with PCa undergoing surgical procedures at the Affiliated Hospital of Youjiang Medical University for Nationalities from May 2022 to April 2023. Plasma specimens were acquired from the aforementioned patients before surgery as well as another group of eight healthy donors. Approval for current study was granted by the ethics committee of the hospital.

### Cell culture and transfection

The human pancreatic ductal epithelial cell (HPDE6-C7) and PCa cell lines (AsPCa-1, BxPCa-3, Capan-1, PANC-1) were acquired from National Cell Centre (Shanghai, China). For cell culture, the cell lines were maintained in DMEM provided by Gibco (USA). The culture conditions were set to a standard temperature of 37°C and an atmosphere containing 5% CO_2_.

The mimic and inhibitor of miR-125a-5p, small interfering RNA (siRNA) against STAT3 (siRNA-STAT3), plasmids for STAT3, along with matching negative controls, were acquired from GenePharma (Shanghai, China). The culture of PANC-1 cells was advanced to achieve an 80% confluence in six-well plates, at which point they underwent transfection with a lentiviral vector following the protocol provided by GenePharma. Transfected cells were then harvested 48 hours subsequent to the transfection process for further evaluation, with gene expression quantified by RT-qPCR.

### Xenograft model in nude mice

Approval for the utilization of nude mice in experimental models was granted by the Institutional Animal Ethics Committee of the Affiliated Hospital of Youjiang Medical University for Nationalities. Nine 5-week-old BALB/c-nu mice, supplied by Cyagen (Suzhou, China), were randomly allocated into three groups: control, miR-125a-5p mimic, and miR-125a-5p inhibitor groups. PANC-1 cells, including untreated and those stably modified with analogue or inhibitor for miR-125a-5p, were subjected to subcutaneous injections into the right axilla of mice. Each mouse received a 0.2 mL injection at a density of 1 × 10^7^ cells/mL in saline solution. Weekly measurements of tumor size were made using vernier calipers, with the volume determined by the equation (mm^3^): volume = length × width^2^ × 0.5. Upon reaching day 28, all mice underwent euthanasia through cervical dislocation, and their tumors were excised for the documentation of volume and weight. Quantification of miR-125a-5p expression in xenograft model was carried out via RT-qPCR.

### TUNEL-based apoptosis assessment

Xenograft tumor tissues embedded in paraffin were sectioned and subsequently deparaffinized. The identification of apoptotic nuclei was achieved using a TUNEL assay kit obtained from Sigma (USA). Following a rinse in PBS, the cells underwent incubation with a streptavidin-horseradish peroxidase (HRP) mixture and a DAB substrate for color development. Cells exhibiting a brown-stained nucleus were classified as TUNEL positive. Photographic documentation of the stained specimens was achieved using a Nikon inverted microscope (Japan). The percentage of TUNEL positive against total cells in the region was calculated using the formula: Apoptosis rate = TUNEL positive cells / total cells × 100%.

### Transmission electron microscopy (TEM)

The xenograft tumor tissues were submerged in a 2.5% solution of glutaraldehyde from Servicebio (China) for a duration of 2 hours at 4℃. Thereafter, the samples underwent dehydration through a sequential ethanol series. Ultrathin slices (80 nm) underwent double staining with uranyl acetate. The resultant photographs depicting autophagosomes were captured using a TEM (Hitachi, Japan) under 80Kv.

### Cell viability

The assessment of cell viability was conducted through the application of the Solarbio Cell Counting Kit-8 (CCK-8; Beijing, China). In this procedure, cells were distributed in 96-well plates, where they were left to grow for 24 hours. Subsequent to transfection, each well was introduced with a 10 μl aliquot of CCK-8 reagent, which was then followed by an additional incubation period (2 hours; 37°C). The quantification of absorbance at 450 nm was performed through the application of a microplate spectrophotometer (Bio-Rad, USA).

### Gene expression quantification *via* RT‑qPCR

The isolation of total RNA from various samples, including tissues, plasma, and cells, was facilitated by the application of Trizol reagent from Invitrogen (USA). Subsequent qPCR steps were executed using the SYBR Green Master Mix kit (Yeasen, China). U6 for miRNA and GAPDH for mRNA served as the housekeeping genes to normalize expression data. The procedures were performed three times for reliability. Gene expression quantification was facilitated through the application of the 2^-ΔΔCt^ calculation method. Primers were as follows: miR-125a-5p: forward 5'-CGTCCCTGAGACCCTTT-3', reverse 5'-CGCTTCACGAATTTGCGTGTCAT-3'; STAT3: forward 5'-GCTGCCCCATACCTGAAGAC-3', reverse 5'-CTCCGAGGTCAACTCCATGTC-3'; U6: forward 5'-GCTTCGGCAGCACATATACTAAAAT-3', reverse 5'-CGCTTCACGAATTTGCGTGTCAT-3'; GAPDH: forward 5′-GGTGGTCTCCTCTGACTTCAA-3′, reverse 5′-GTTGCTGTAGCCAAATTCGTTGT-3′.

### Western botting analysis

Cells and tissues were harvested and subjected to lysis using RIPA buffer (Beyotime, China) under chilled conditions for a duration of 30 min. Afterward, the quantification of protein levels was carried out using the Bicinchoninic Acid (BCA) Protein Assay Kit provided by Epizyme (China). Proteins (30 µg per lane) were subjected to separation through 10% or 12.5% SDS‑PAGE and subsequently electrophoresis procedure. To reduce unspecific interactions, the membranes underwent a 2-hour blocking process in 5% skim milk at ambient temperature. Thereafter, they were exposed to primary antibodies (Abcam, UK) against STAT3, LC3, Beclin-1, Caspase-3, and Caspase-8 (diluted 1:1000 for all except Caspase-8 at 1:500), as well as the loading control β-actin (at a dilution of 1:5000) at 4°C overnight. Secondary antibodies were applied to the membranes the following day. The protein bands were photographed using a chemiluminescent imaging apparatus, and their quantification was facilitated using ImageJ V1.53 software.

### Dual-Luciferase activity assay

The putative binding affinity of miR-125a-5p to STAT3 was predicted utilizing the TargetScan database (accessible at http://www.targetscan.org/). Constructs of both wild- and mutant-type STAT3 mRNA 3′ zone fragments, along with the mimics for miR-125a-5p, were developed and incorporated into plasmid constructs. The cells were planted onto a 24-well plate and subjected to transfection incorporating the designated plasmid and the mimics of miR-125a-5p for a period of 48 hours utilizing the X-tremegene HP transfection reagent provided by Roche (Switzerland). Finally, luciferase activity was detected and quantified.

### Statistical analysis

Kaplan-Meier curves were subjected to analysis using the Log-Rank test. All assays were replicated in no fewer than three experiments. The results were displayed as the mean ± standard deviation (SD) of triplicate experiments. The analysis of experimental data was conducted utilizing GraphPad Prism statistical software (version 9.0). Data from two groups were evaluated using the student t-test, whereas data from multiple groups were subjected to one-way analysis of variance (ANOVA). Results were deemed statistically significant when *P* < 0.05.

## Results

### The expression pattern and prognostic relevance of miR-125a-5p and STAT3 on PCa

**Figure 1:**
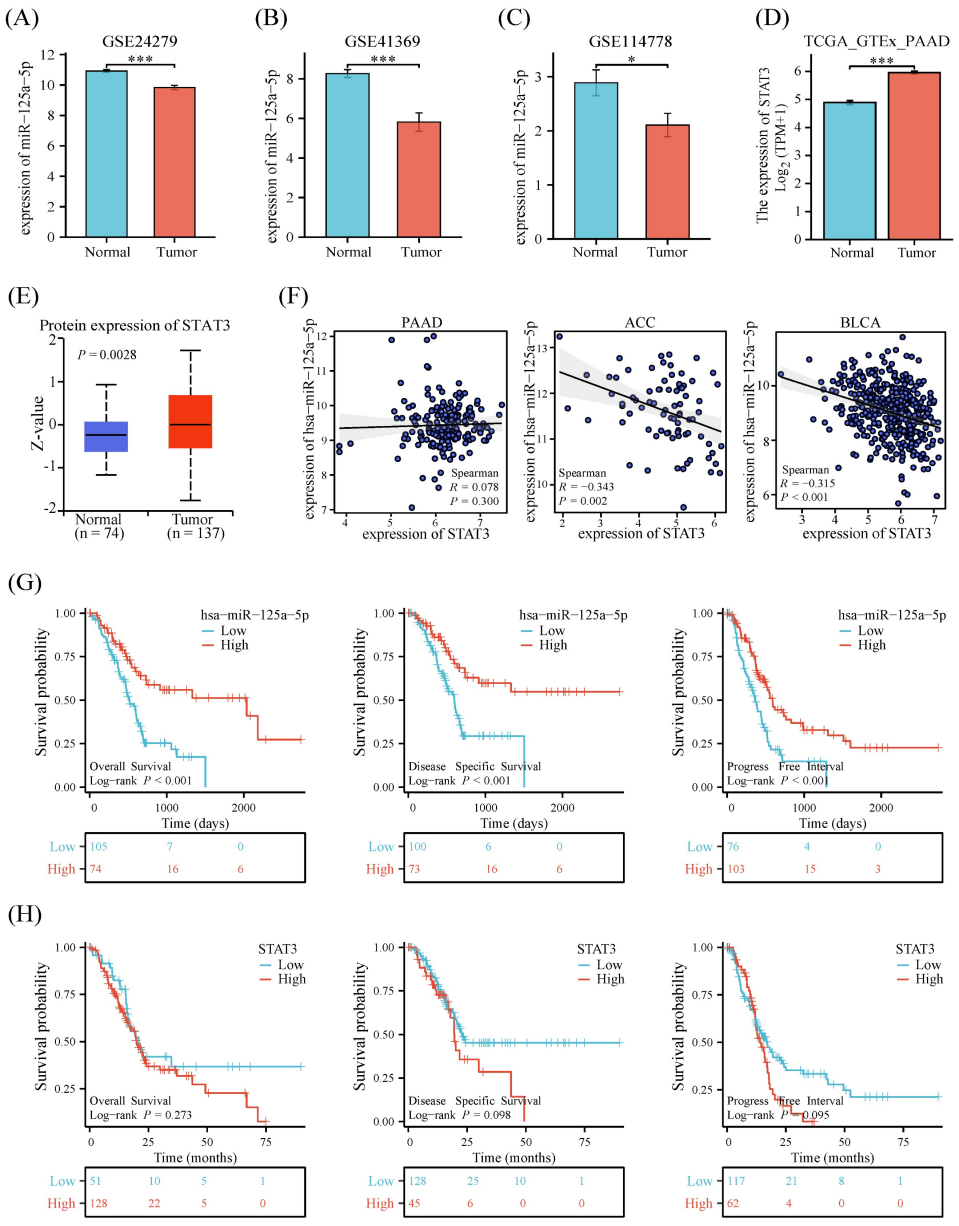
The expression pattern and prognostic relevance of miR-125a-5p and STAT3 on PCa. The expression of miR-125a-5p was significantly decreased in PCa patients than that of healthy controls in (**A**) GSE24279, (**B**) GSE41369, and (**C**)GSE114778. (**D**) The mRNA expression and (**E**) protein expression level of STAT3 in PCa tissues and non-tumor tissues from GTEx. (**F**) Correlation of miR-125a-5p and STAT3 expression levels in PAAD, ACC, and BLCA. (**G**) The Kaplan-Meier survival plots, including overall survival, disease-specific survival, and progression-free interval were used to evaluate the prognostic value of miR-125a-5p expression in PCa. The high expression of miR-125a-5p suggested a better prognosis. (**H**) The Kaplan-Meier survival plots of STAT3 expression in PCa. PAAD: pancreatic adenocarcinoma. ACC: adrenocortical carcinoma, BLCA: bladder urothelial carcinoma. *P < 0.05, ***P < 0.001.

Examination of three datasets (GSE24279, GSE41369, and GSE114778) unveiled a notable downregulation (*P* < 0.05) in miR-125a-5p levels in the PCa group when contrasted with healthy controls (Figure 1A-C). Both mRNA and protein expression levels of STAT3 were statistically significantly decreased in PCa and non-tumor tissues from the Genotype-Tissue Expression Project (GTEx) and TCGA (Figure 1D-E). To explore the relationship between miR-125a-5p and STAT3 expression, we analyzed the expression profiles of both. The results showed no correlation between miR-125a-5p and STAT3 expression in pancreatic adenocarcinoma (PAAD), however, in adrenocortical carcinoma (ACC) and bladder urothelial carcinoma (BLCA), there was a significant negative correlation between miR-125a-5p and STAT3 expression (Figure 1F). To delve into the potential link between miR-125a-5p and STAT3 expression levels and prognostic outcomes of PCa, we retrieved miRNA and mRNA transcriptomic data from the TCGA database as well as clinical information on PCa patients. Higher expression of miR-125a-5p demonstrated positive correlation with better prognostic parameters (Figure 1G). Although there was no statistical correlation between STAT3 expression and PCa prognosis, a trend towards higher expression of STAT3 leading to a poorer prognosis could be observed (Figure 1H).

### Pan-cancer analysis of STAT3 and validation of miR-125a-5p expression

**Figure 2:**
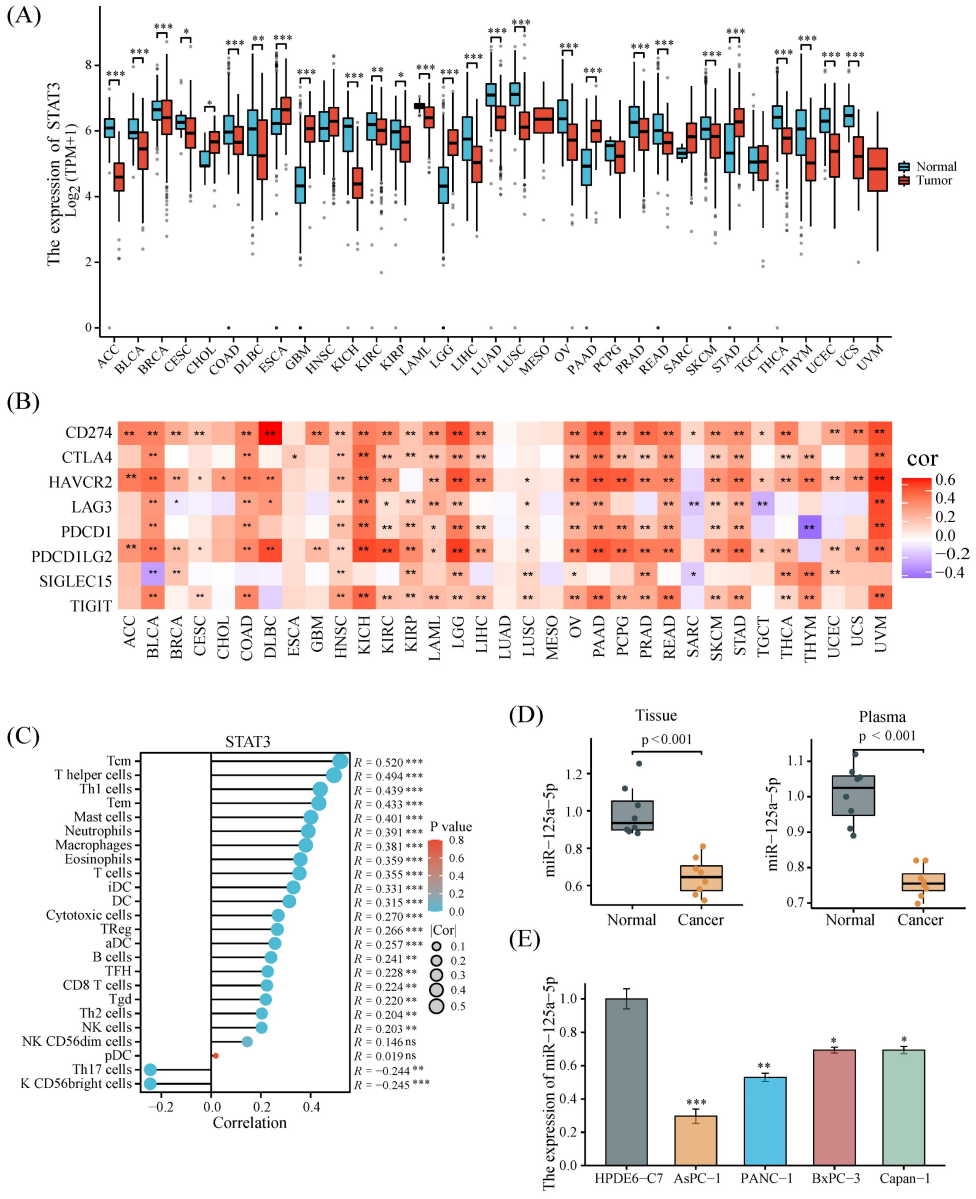
Pan-cancer analysis of STAT3 and validation of miR-125a-5p expression. (**A**) STAT3 expression levels in 33 kinds of cancer based on the TCGA database. (**B**) Correlation analysis between STAT3 expression and immune checkpoints. (**C**) Correlation analysis of STAT3 expression with immune cells in PCa. (**D**) The mRNA expression level of miR-125a-5p in tissue and plasma of PCa patients and healthy donors in our collected cases. (**E**) miR-125a-5p expression in PCa cell lines was significantly downregulated than that in HPDE6-C7 cells. *P < 0.05, **P < 0.01, ***P < 0.001.

A pan-cancer analysis combining data from the TCGA and GTEx databases uncovered that STAT3 was significantly highly expressed in only a few tumors, particularly in PCa (Figure 2A). We also sought to understand the correlation between STAT3 expression and immune resistance. Figure 2B demonstrated that STAT3 expression was positively correlated with immune checkpoints in most tumors. The correlation between STAT3 expression and the infiltration of multiple immune cells indicated its possible involvement in the regulation of the tumor immune microenvironment (Figure 2C). The expression pattern of miR-125a-5p in PCa tumour tissues and plasma samples was subsequently corroborated utilizing our independent cohort. More clinical information of the cohort is shown in Supplementary Table S1. The results revealed a pronounced decline in miR-125a-5p expression in PCa tumor tissues, as well as plasma when compared to their respective controls (*P* < 0.001, Figure 2D). Moreover, the examination of miR-125a-5p levels in PCa cell lines uncovered a prominent downregulation (*P* < 0.05) in miR-125a-5p expression in these cell lines relative to HPDE6-C7 cells (Figure 2E). Given the moderate expression of miR-125a-5p, PANC-1 cells were chosen as suitable candidates for gene overexpression or silencing investigations.

### miR-125a-5p hinders proliferation, increases autophagy and apoptosis *in vitro*

**Figure 3:**
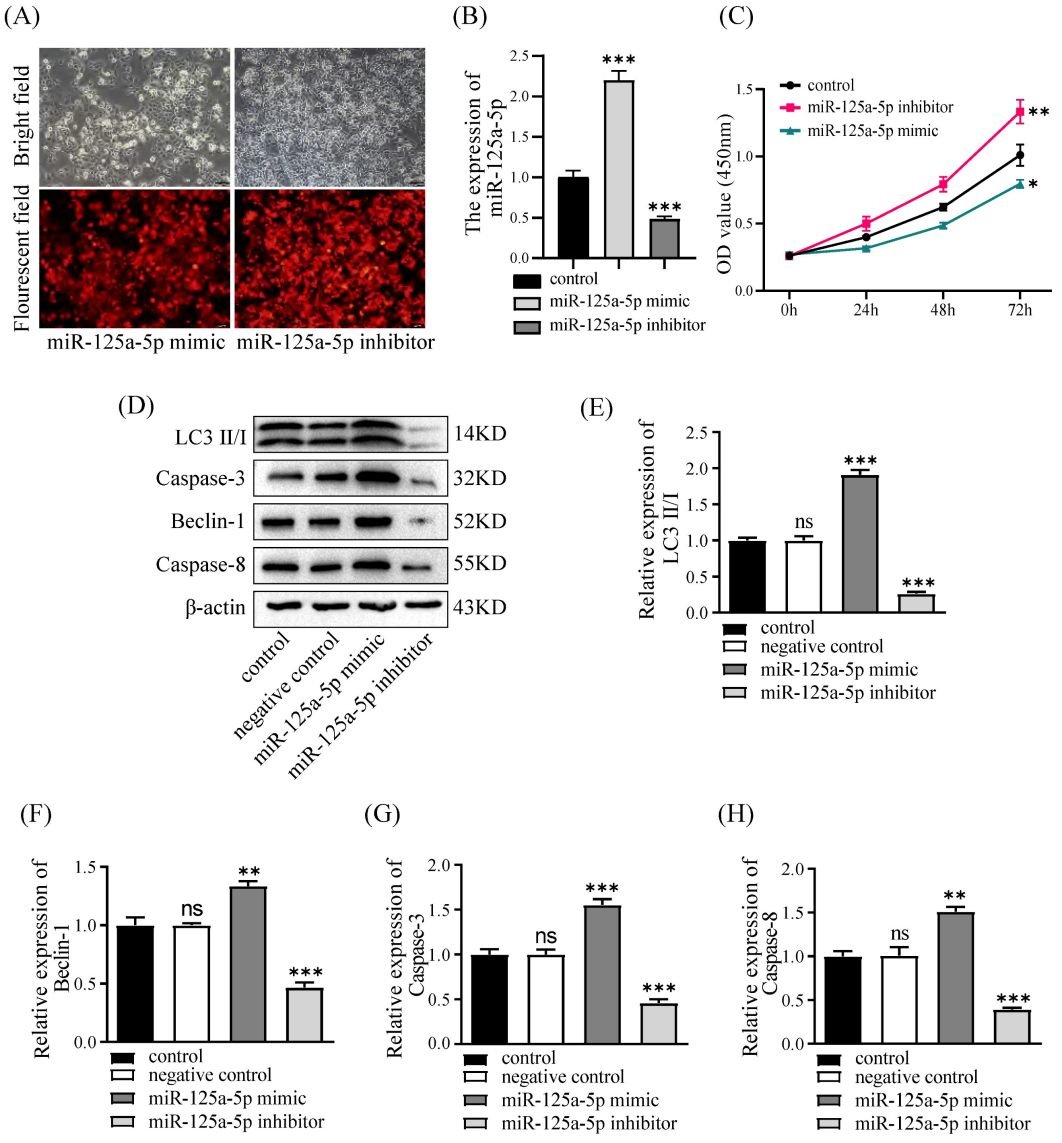
miR-125a-5p inhibited proliferation, increased autophagy and apoptosis *in vitro.* (**A**) miR-125a-5p mimic and inhibitor were transfected into PANC-1cells with the fluorescent protein gene, and the transfection efficiency can be determined by fluorescence microscopy. (**B**) The transfection efficiency was verified by RT-qPCR. (**C**) CCK-8 assay was performed to determine cell proliferation of PANC-1 transfection with miR-125a-5p mimic or miR-125a-5p inhibitor at 24, 48 and 72 hours. (**D**) The expression of autophagy- and apoptosis-related proteins, including LC3 (**E**), Beclin-1 (**F**), Caspase-3 (**G**), and Caspase-8 (**H**) were detected. ns: not statistically significant, *P < 0.05, **P < 0.01, ***P < 0.001.

To substantiate the functional relevance of miR-125a-5p, PANC-1 cell lines underwent transfection with the mimic or inhibitor of miR-125a-5p, followed by the assessment of miR-125a-5p levels. The lentiviral transfection efficiencies of the miR-125a-5p mimic and inhibitor groups were satisfactory, with both exceeding 90% of cells being transfected with lentivirus (Figure 3A). Notably, the RT-qPCR results revealed a significant augmentation or reduction inmiR-125a-5p expression subsequent to the delivery of its mimic or inhibitor (*P* < 0.001, Figure 3B). Assessment of cell viability was conducted through the CCK-8 experiments. Interestingly, ectopic expression of miR-125a-5p caused pronounced decrease in cell viability, whereas its inhibition prominently enhanced activity of PANC-1 cells (*P* < 0.05, as depicted in Figure 3C). Furthermore, Western blot results demonstrated elevation of miR-125a-5p expression facilitated autophagy- and apoptosis-related proteins, including LC3, Beclin-1, Caspase-3, and Caspase-8 (Figure 3D-H).

### miR-125a-5p suppresses tumor progression of PCa *in vivo*

**Figure 4:**
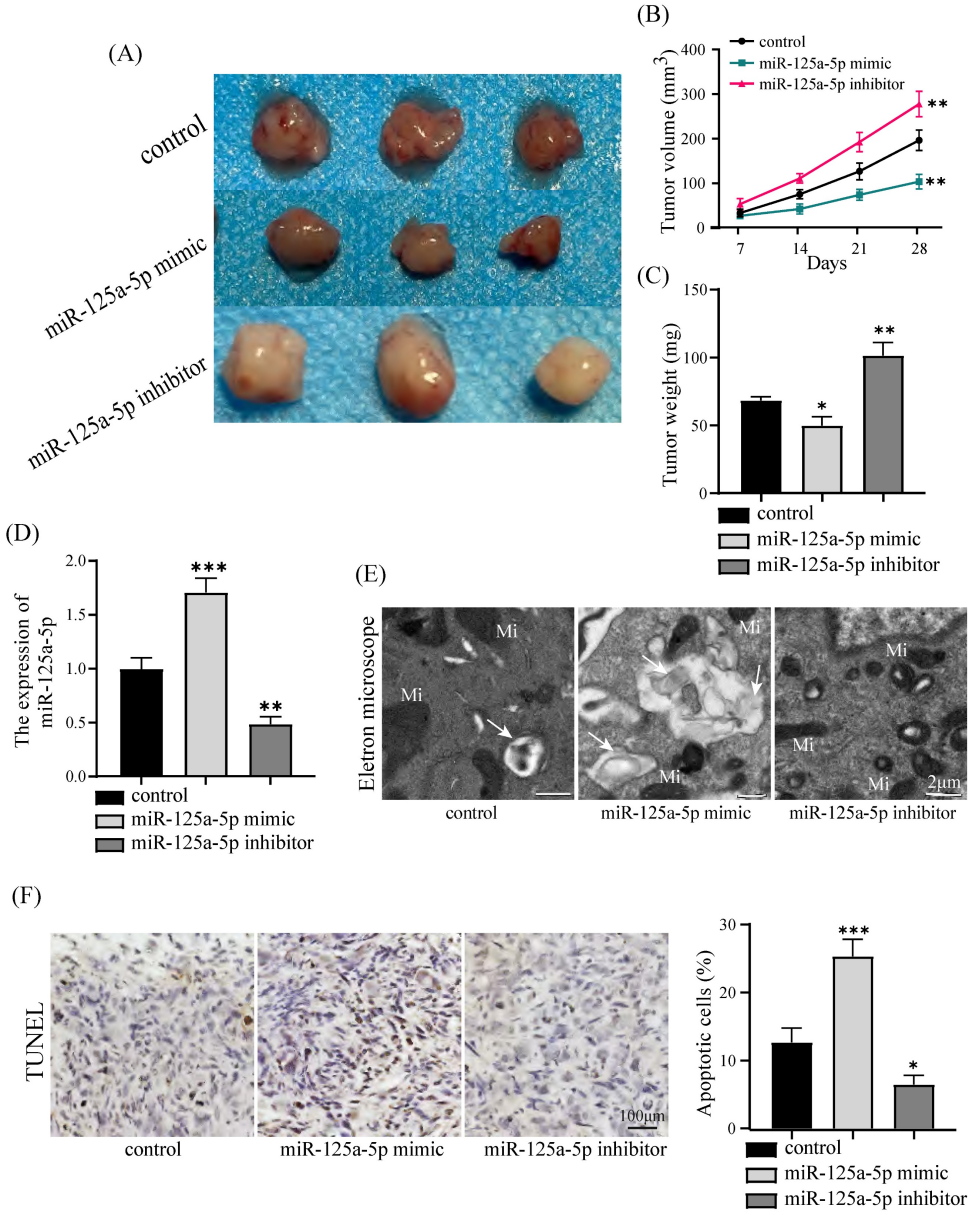
miR-125a-5p overexpression suppressed PCa tumor growth *in vivo.* (**A**) Gross images of tumor sizes in control (PANC-1 cell), miR-125a-5p mimic, and miR-125a-5p inhibitor groups. (**B**) Tumor volumes at 7, 14, 21, and 28 days in a xenograft model that was injected subcutaneously with PANC-1 cells. (**C**) Tumor weights of control, miR-125a-5p mimic and miR-125a-5p inhibitor groups were compared. (**D**) Expression of miR-125a-5p in xenograft tumor tissues was detected using RT-qPCR. (**E**) Autophagosome formation in tumor tissues was captured by transmission electron microscopy. Autophagosome (→), mitochondria (Mi). (**F**) TUNEL assay was performed to detect the apoptosis and the percentage of apoptotic cells was compared in three different groups. *P < 0.05, **P < 0.01, ***P < 0.001.

**Figure 5:**
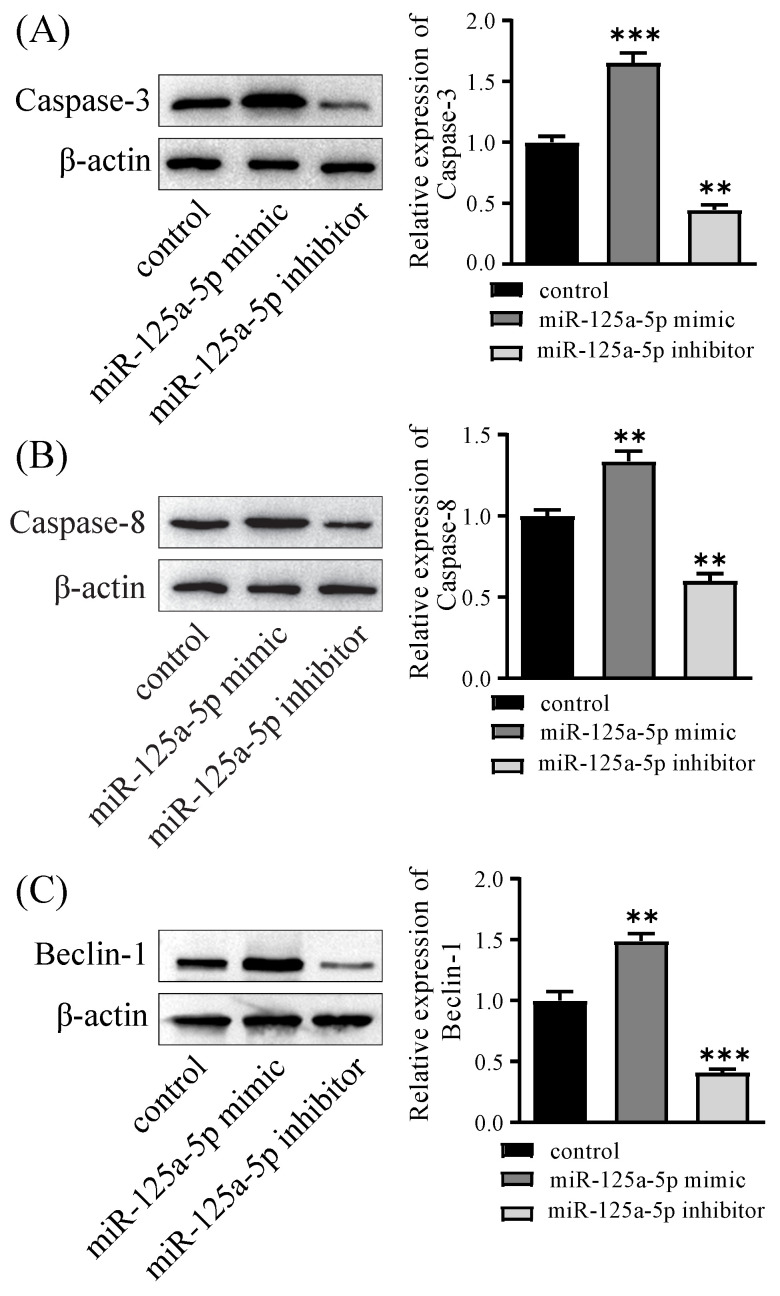
The overexpression of miR-125a-5p in a xenograft model increased the expression of autophagy- and apoptosis-related proteins, including Caspase-3 (**A**), Caspase-8 (**B**), and Beclin-1 (**C**). **P < 0.01, ***P < 0.001.

PANC-1 cells were injected to nude mice. A xenograft tumor model was established in nude mice to substantiate the potential antitumor impact of miR-125a-5p on PCa. It is worth noting that miR-125a-5p mimic treatment resulted in significant reductions in both the size and weight of tumors. This indicates the potential effectiveness of miR-125a-5p mimic in suppressing tumor growth, as observed in inhibitor and control groups (*P* < 0.05, Figure 4A-C). In addition, in the miR-125a-5p mimic group, there was a substantial increase in the expression of miR-125a-5p compared to both the miR-125a-5p inhibitor and control groups (*P* < 0.01, Figure 4D). The observations through transmission electron microscopy unveiled the repression of miR-125a-5p expression hindered autophagosome formation, resulting in autophagy deficiency (Figure 4E). Elevated expression of miR-125a-5p was demonstrated to enhance apoptosis as demonstrated by TUNEL assay results (*P* < 0.05, Figure 4F). Moreover, Elevated level of miR-125a-5p increased transcription of autophagy and apoptosis-related proteins in tumor tissues (*P* < 0.01, Figure 5A-C). Cumulatively, these findings suggest the antioncogenic activity of miR-125a-5p on PCa *in vivo* by diminishing proliferation while augmenting autophagy and apoptosis.

### miR-125a-5p directly targets STAT3, which is downregulated in PCa tissues

**Figure 6:**
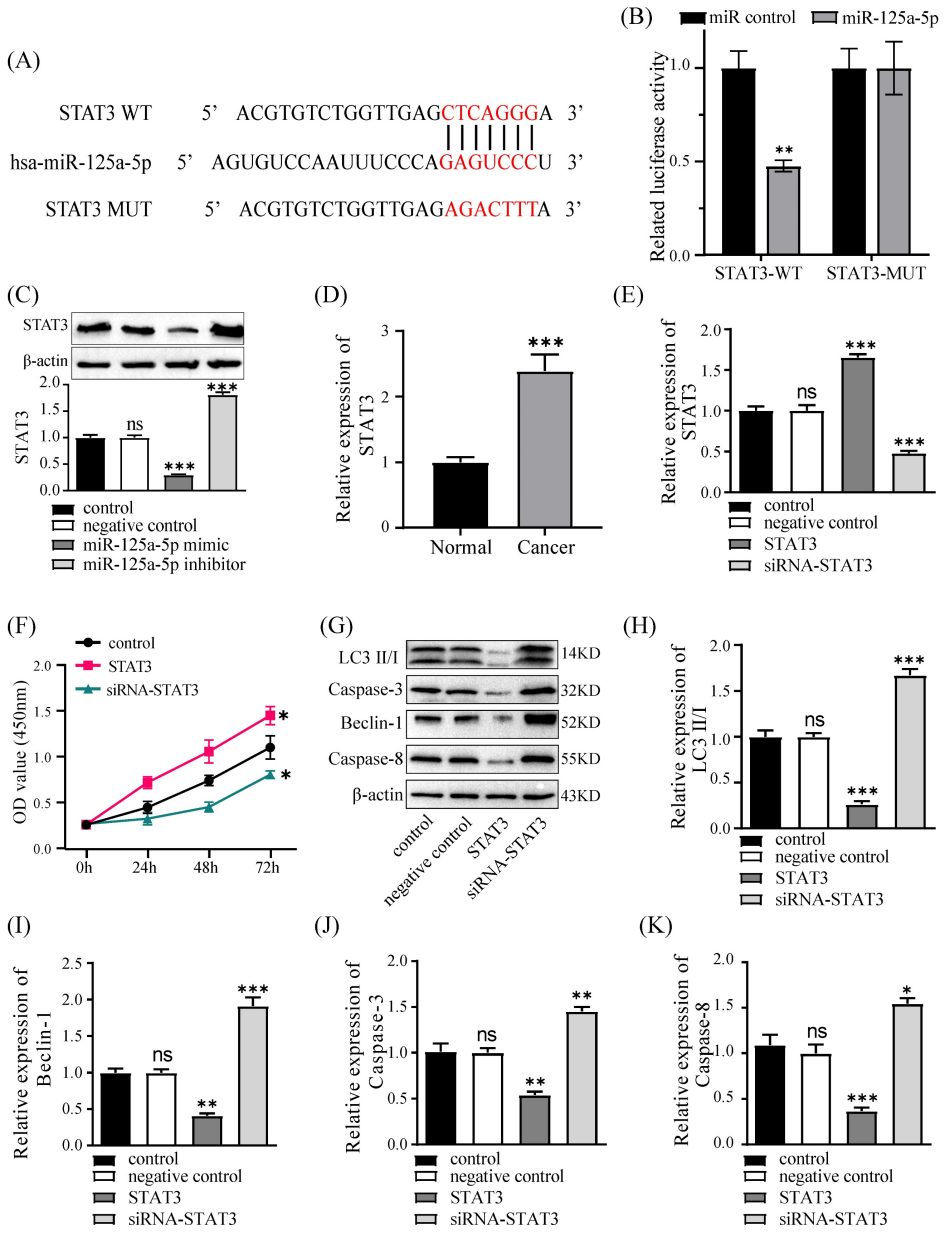
STAT3 is a target gene of miR-125a-5p, it promoted cell proliferation and inhibited autophagy and apoptosis. (**A**) TargetScan database predicted the binding site between miR-125a-5p and STAT3 3'-UTR. (**B**) The luciferase reporter plasmid with wild-type (WT) and mutant type (MUT) STAT3 was co-transfected with miR-125a-5p mimic or miR control. Related luciferase activity was measured 48 hours after transfection. (**C**) STAT3 protein expression was detected after transfection with miR-125a-5p mimic, miR-125a-5p inhibitor and negative control. (**D**) The relative mRNA expression of STAT3 was significantly upregulated in PCa tissues than that in adjacent normal tissues. (**E**) STAT3 protein expression was detected after transfection with siRNA-STAT3, STAT3 (overexpressing STAT3) and negative control. (**F**) CCK-8 assay was performed to determine cell proliferation of PANC-1 transfected with miR-125a-5p mimic or miR-125a-5p inhibitor for 24 h, 48 h, and 72 h. (**G**) The expression of LC3 (**H**), Beclin-1 (**I**), Caspase-3 (**J**), and Caspase-8 (**K**) was detected after transfection with siRNA-STAT3, STAT3 and negative control. ns: not statistically significant, *P < 0.05, **P < 0.01, ***P < 0.001.

The TargetScan database predicted that miR-125a-5p could connect to the 3' zone of STAT3 (Figure 6A). Subsequent validation uncovered the miR-125a analogue notably restricted luciferase activity of wild-type STAT3, whereas it had no effect on mutant STAT3. (*P* < 0.01, Figure 6B). In PANC-1 cells, miR-125a-5p was observed to suppress STAT3 expression, and a negative correlation between them was established (*P* < 0.001, Figure 6C). Furthermore, to elucidate the involvement of STAT3 in PCa, the levels of STAT3 were evaluated in tumor tissues from patients with PCa, revealing a noteworthy upregulation of STAT3 expression in PCa tissues (Figure 6D). Subsequently, we managed to establish PANC-1 cell lines with stable overexpression or silencing of STAT3 (Figure 6E). The CCK-8 assay findings underscored a noteworthy augmentation in cell viability upon STAT3 overexpression (Figure 6F). Furthermore, our investigation delved into the regulatory role of STAT3 on autophagic and apoptotic capacities in PANC-1 cells, demonstrating that STAT3 restricted the levels of autophagy- and apoptosis-related proteins, including LC3, Beclin-1, Caspase-3, and Caspase-8 (Figure 6G-K).

### Silencing of STAT3 counteracts the impacts of miR-125a-5p inhibition

**Figure 7:**
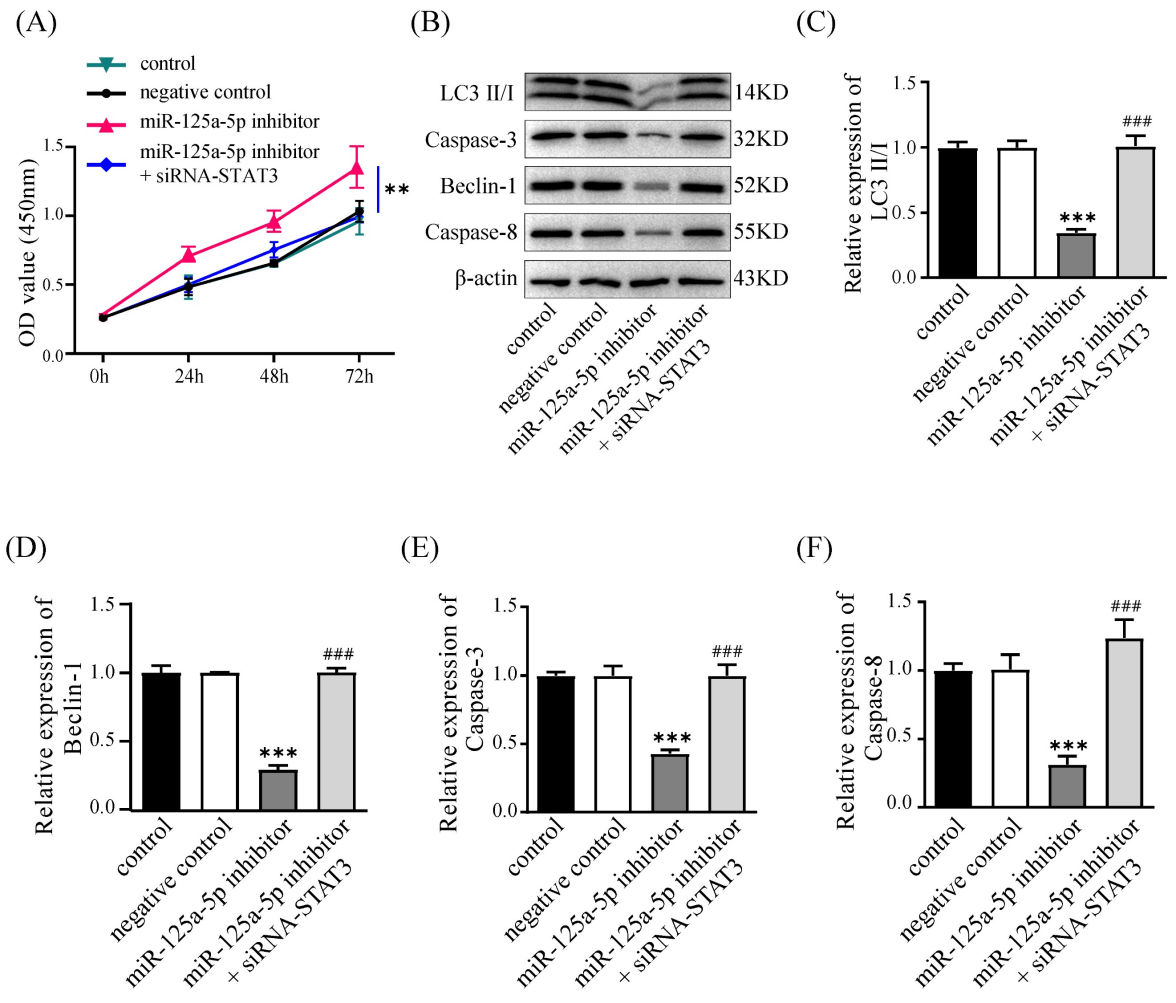
Knockdown of STAT3 could reverse the effects of miR-125a-5p inhibition. (**A**) Cell proliferation was measured by CCK-8 assay after transfection. (**B**) The expression of LC3 (**C**), Beclin-1 (**E**), Caspase-3 (**E**), and Caspase-8 (**F**), was detected after transfection. *P < 0.05, **P < 0.01, ***P < 0.001 vs. control. ###* P* < 0.001 vs. miR-125a-5p inhibitor group.

We conducted rescue experiments in PANC-1 cells, assessing cell proliferation, autophagy, and apoptosis to validate the regulatory link of miR-125a-5p with STAT3. Suppressing miR-125a-5p led to elevated STAT3 expression levels and promoted cell proliferation at an accelerated rate; in contrast, silencing of STAT3 counteracted this effect and hindered cell proliferation (Figure 7A). Furthermore, miR-125a-5p inhibition curbed the levels of autophagy- and apoptosis-related proteins; however, silencing of STAT3 reversed this effect, suggesting that miR-125a-5p likely exerts its biological functions through targeting STAT3 (*P* < 0.001, Figure 7B-F).

### miR -125a-5p fosters PCa cell apoptosis via targeting STAT3

**Figure 8:**
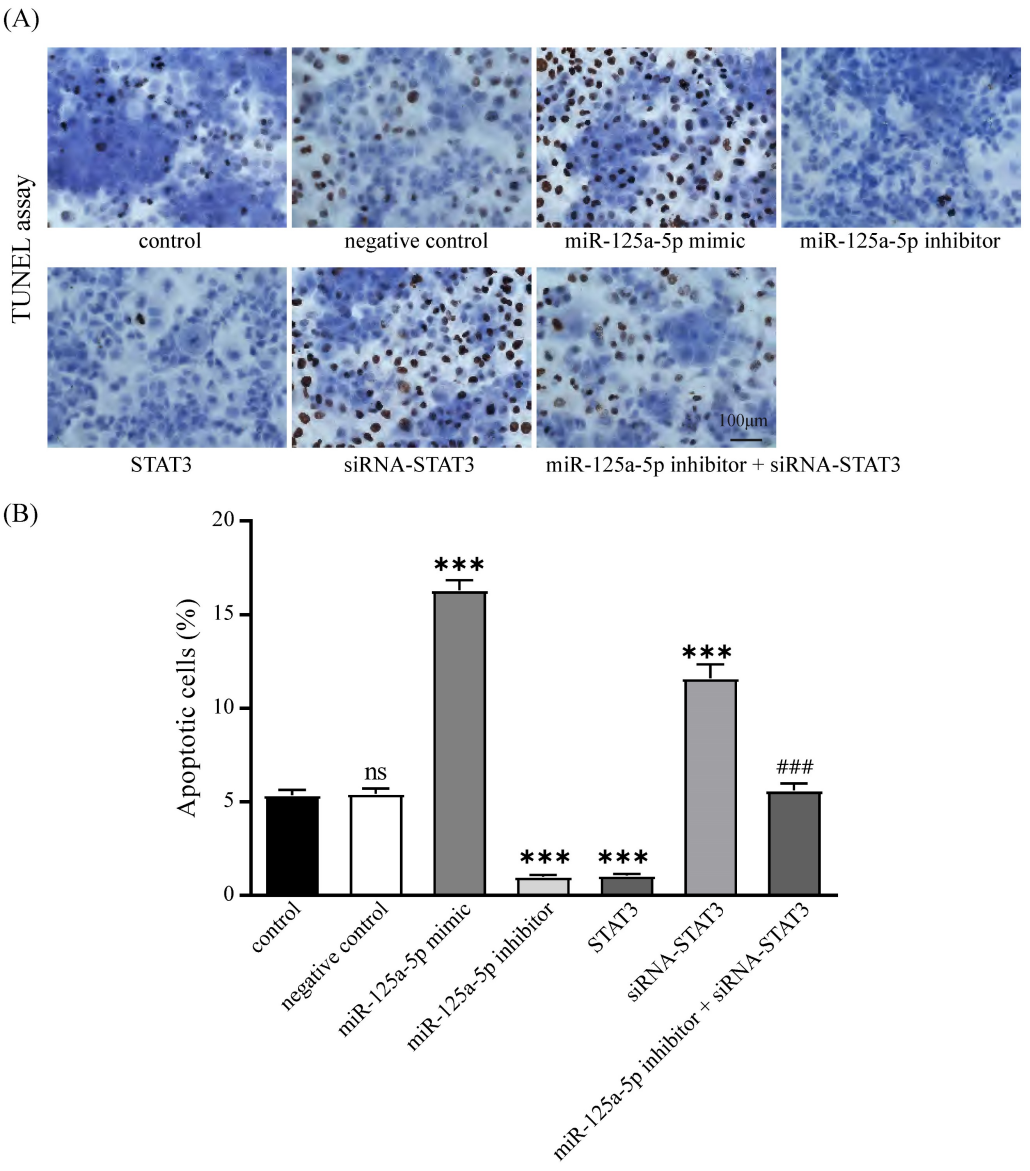
miR-125a-5p promoted apoptosis by targeting STAT3. (**A**) TUNEL assay was performed to detect the apoptosis of PANC-1 cells and the percentage of apoptotic cells was compared (**B**). ns: not statistically significant, ***P < 0.001 *vs.* control. ###* P* < 0.001 *vs.* miR-125a-5p inhibitor group.

The impact of miR-125a-5p and STAT3 on apoptosis in PANC-1 cells was corroborated through TUNEL-based assays. The results delineated that miR-125a-5p facilitated apoptosis, while STAT3 hindered this process. Additionally, concurrent suppression of miR-125a-5p and STAT3 resulted in a marked increase in apoptosis relative to miR-125a-5p inhibitor alone (Figure 8A-B). These findings collectively imply that miR-125a-5p attenuates the apoptotic potential by targeting STAT3.

## Discussion

In a variety of cancers, miRNAs have been attributed with oncogenic properties that promote tumor growth or suppressive properties that inhibit tumor development^19^. miRNAs have been a research hotspot in recent years, and their application as biomarkers in the diagnosis and treatment of PCa has received increasing attention^20^. Several investigations have provided promising insights into the therapeutic potential of miRNAs for managing PCa^21^. Specifically, diminished levels of miR-454, miR-506-3p, miR-221, and miR-181a have been observed with the progression of PCa^21^-^24^, whereas miR-600 exhibits an upregulated expression profile^25^. Diminished miR-125a-5p expression has been identified in various cancer types, suggesting its involvement in cancer development and progression^26^. Consistent with prior investigations, our findings corroborate the underexpression of miR-125a-5p in PCa tissues, plasma samples, and PCa cell lines, thereby indicating a potential association with PCa. Manipulation of miR-125a-5p levels in PANC-1 cells elicited notable outcomes, as heightened miR-125a-5p expression hampered cell growth activity and bolstered autophagy and apoptosis processes, as evidenced by upregulated levels of LC3, Beclin-1, Caspase-3, and Caspase-8 proteins. In addition, the augmentation of miR-125a-5p expression curbed tumour progression of PCa in nude mouse xenograft model.

In tumorigenesis and progression, miRNAs primarily function by connecting to the 3' zone of its target genes, thereby modulating the post-transcriptional levels of target genes^4^. The prediction of potential target for miR-125a-5p was achieved from the TargetScan database in our investigation, which found STAT3 as one of its targets. Subsequently, this regulatory binding affinity was validated through dual-luciferase reporter assays. Based on bioinformatics analysis, we found that STAT3 is differently expressed between tumor tissues and normal tissues. What's more, STAT3 is positively correlated with the expression of immune checkpoint genes and the infiltration of immune cells. STAT3 is believed to be involved in the regulation of the tumor microenvironment. Better elucidation of the biological secrets of the tumor microenvironment will contribute to a more precise immunotherapy of tumors^27^. STAT3 is necessary for T-cell activation, and the activation of STAT3 suppresses immune cells and remodels T-cell subtype ratios^28^. Combination therapies that integrate STAT3 inhibitors and immune checkpoint inhibitors may contribute to the discovery of more effective clinical therapeutic treatments^29^, ^30^. STAT3 has been identified as a pivotal transcription factor, playing an essential and critical role in the processes of carcinogenesis and the development of drug resistance^15^. Chen H *et al.* have illuminated that STAT3 regulates the expression of TNFAIP3, a gene implicated in gemcitabine resistance in PCa. Consequently, the strategic targeting of STAT3 holds significant promise as a viable approach to counteract drug resistance in PCa^31^. Ming W *et al.* have found that STAT3 exhibits the property of promoting tumor progression, and blocking the STAT3 pathway can enhance the apoptosis and autophagy of glioma cells, which can lead to a better clinical therapeutic effect^32^. Zhao Y *et al.* conducted a study in which they revealed that miR-125a-5p enabled esophageal squamous cell carcinoma cells to exhibit higher sensitivity against cisplatin treatment by modulating STAT3 expression, thereby contributing to the enhancement of treatment efficacy for this type of cancer^17^. Immune checkpoint inhibitors are promising anti-tumor therapies, and their combination with conventional chemoradiotherapy improves anti-tumor efficacy and increases the survival time of patients^28^. An in-depth study of the potential mechanisms of STAT3 inhibitors in PCa could accelerate their clinical application.

In addition, existing studies have documented the upregulation of STAT3 in breast and lung cancers, which can be restricted by miR-125a-5p^8^. In our study, it was observed that the expression levels of STAT3 exhibited a marked increase in PCa tissues as opposed to the levels detected in the surrounding normal tissues. *In vitro* assays revealed that STAT3 overexpression bolstered cell proliferation and diminished autophagy and apoptosis, thereby counteracting the antioncogenic effects elicited by miR-125a-5p.

Autophagy is a biological process related to cell death and metabolism, and contributes to the decomposition of damaged intracellular substances and the recycle of metabolites^33^. In the early stages of tumorigenesis, autophagy can suppress tumors by degrading potentially oncogenic molecules. For example, miR-23b has been shown to regulate autophagy in PCa cells by targeting ATG12^34^. The study of Yan L *et al.* unveiled the autophagy-enhancing capacity of miR-21-5p *via* activating the PTEN/AKT signaling in skin wound healing^35^. Our findings align with this by demonstrating that miR-125a-5p augmented autophagic potential in PCa cells and xenograft tumor growth. This finding supports the role of miRNAs in autophagy dynamics regulation within the context of PCa, aligning with suggestions from previous research. Understanding the multifaceted role of autophagy in cancer requires in-depth investigation across different cancer types and stages to decipher the specific effects of autophagy modulation.

However, the study had certain limitations. One such constraint was the limited number of clinical samples available, which restricted a comprehensive analysis of how miR-125a-5p and STAT3 correlate with clinicopathological variables and prognostic outcomes in patients with PCa. This issue is anticipated to be resolved promptly with the ongoing collection of additional PCa cases. Moreover, a growing body of research underscores the pivotal involvement of exosomes in antitumor and stem cell therapies, prompting our future endeavors to elucidate the regulatory pathways modulated by exosome-delivered miR-125a-5p in PCa.

## Conclusions

In summary, our investigation elucidates the function of miR-125a-5p in impeding cell growth and promoting autophagy and apoptosis in PCa cells by targeting STAT3. Current study highlights the prospect of miR-125a-5p as a promising target for prognostic and therapeutic strategies in PCa.

## Supplementary Material

Supplementary table.

## Abbreviations

PCa: pancreatic cancer
miR-125a-5p: MicroRNA-125a-5p
STAT3: signal transducer and activator of transcription 3
GEO: Gene Expression Omnibus
TCGA: The Cancer Genome Atlas
GTEx: Genotype-Tissue Expression Project
TUNEL: TdT-mediated dUTP nick end labeling
