# Supplementary material for: Unlocking the Therapeutic Potential: Harnessing miR-125a-5p To Enhance Autophagy and Apoptosis in Pancreatic Cancer through Targeting STAT3
Source: J Cancer. 2024 Jul 16;15(15):4955–68. doi: 10.7150/jca.97102 (PMC11310883; doi:10.7150/jca.97102)
Supplement: Supplementary file 1 — Supplementary table. [file jcav15p4955s1.pdf]

Supplementary table S1 Baseline tumor characteristics

Characteristic	N = 8
Age (min ~ max)	46 ~ 72
Gender	
Male	3
Female	5
Histology	
Adenocarcinoma	8
Location of lesion	
Pancreatic head	5
Body	2
Tail	1
Stage T	
T1	1
T2	3
T3	3
T4	1
Stage N	
N0	5
N1	3
Stage M	
M0	8
Size of tumor	
Mean ± SD	3.2 ± 1.2
Min, max	0.6, 5.8

Note: Abbreviations: max = maximum; min = minimum; SD = standard deviation; TNM = tumor, node, metastases.
